# Health disinformation & social media: The crucial role of information hygiene in mitigating conspiracy theory and infodemics

**DOI:** 10.15252/embr.202051819

**Published:** 2020-11-05

**Authors:** David Robert Grimes

**Affiliations:** ^1^ School of Physical Sciences Dublin City University Dublin Ireland; ^2^ Department of Oncology University of Oxford Oxford UK

**Keywords:** S&S: Economics & Business, S&S: Ethics

## Abstract

Social media has been an effective vector for spreading disinformation about medicine and science. Informational hygiene can reduce the severity of falsehoods about health.

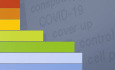

The satirist Johnathan Swift once lamented that “falsehood flies, and truth comes limping after it”. Penned three centuries ago, these words seem alarmingly prescient: The paradox of the era we live in is that, despite having access to an enormous amount of information at our fingertips, this same freedom allows poisonous fictions to aggressively perpetuate. In the midst of the COVID‐19 pandemic, this has manifested as a parallel crisis of information—in the words of the WHO, we are experiencing “an overabundance of information, some accurate and some not, that makes it hard for people to find trustworthy sources and reliable guidance when they need it”. In this, the first pandemic we face in the social media era, we are glimpsing the rise of a shadow problem—an “infodemic”, the dissemination of disinformation across social media.

In this, the first pandemic we face in the social media era, we are glimpsing the rise of a shadow problem—an “infodemic”…

## The viral nature of disinformation

Claims of dubious veracity frequently go viral on the internet. This pathogenic allegory for modern information is not idly made. We all too frequently act as vectors for fictions, which can cause societal harm. In the UK and beyond, conspiracy theories linking 5G communications to COVID‐19 saw cell towers torched by arsonists. But falsehoods about radiofrequency are not new—current assertions about ostensible harms of 5G are simply recycled from prior scaremongering about cell‐phones, WiFi and powerlines, and bereft of any substance (Grimes & Bishop 2018).

And while this might be a novel coronavirus, the conspiracy theories flaunted around it are anything but long before YouTube cranks insisted COVID‐19 was a man‐made virus, claims that AIDS was a CIA bioweapon gained traction in the 1980s. On this latter point, the fingerprints of Russian and Chinese disinformation underpinning current Coronavirus myths have historical echoes; at the height of the AIDs crisis, Soviet intelligence invested heavily in amplifying the claims the disease was an American weapon. This undertaking, the notorious operation INFEKTION, was ultimately derailed when the pandemic swept the USSR, forcing Russia to seek help from American virologists. Such a spectacular volte‐face underpins an inescapable truth: Reality does not care one iota for our narratives. And despite being debunked for decades, the myth still maintains significant traction.

**Figure 1:**
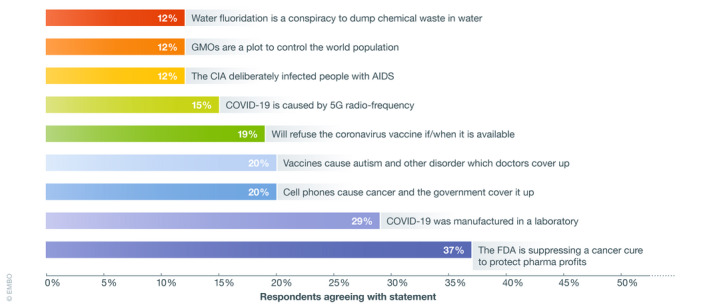
Commonly encountered conspiratorial beliefs in medicine Estimated proportion of population subscribing to common medical conspiracy theories. Based on data from Oliver and Wood (2014) and surveys from the Université de Sherbrooke (https://www.usherbrooke.ca/actualites/relations-medias/communiques/communiques-details/c/42738/) and the Pew Research Center (https://www.pewresearch.org/fact-tank/2020/07/24/a-look-at-the-americans-who-believe-there-is-some-truth-to-the-conspiracy-theory-that-covid-19-was-planned/).

More recently, the rise of “anti‐mask” protests has seen disparate groups from anti‐vaccine activists to 5G protesters to alternative health advocates unite under a single banner. Lockdown restrictions are the chief target of their ire. Masks, they claim, lead to oxygen depletion and carbon dioxide poisoning. That this is easily refuted by simple measurement or by observing the army of healthcare workers using them without ill‐effect has been no impediment to the propagation of such myths. These are not insubstantial gatherings. August saw more than 17,000 protestors take to the streets of Berlin alone, with thousands more in London, Dublin, Madrid, and elsewhere. But while the humble mask might be the ostensible focal point, the astute observer cannot fail to notice the banners replete with all manner of health conspiracy, ranging from assertions that the virus is a hoax to the claim we are being microchipped by vaccine manufacturers or Bill Gates (Fig 1).

…, the rise of “anti‐mask” protests has seen disparate groups from anti‐vaccine activists to 5G protesters to alternative health advocates unite under a single banner.

## The grand health conspiracy

In normal times, it is easy to dismiss health conspiracies as a phenomenon confined solely to the fringes, inconsequential for most of us. But the sheer dominance of disinformation in science and medicine should disabuse us of the comfortable notion; this is something we can disregard—and that this has been a serious issue long before the emergence of COVID‐19. This is perhaps most stark when we consider the resurgence in vaccine‐preventable illness. In 2000, measles was declared eradicated in the USA. Twenty years later, the situation has deteriorated alarmingly, with record outbreaks recorded around the country. In 2018, Europe recorded 84,462 cases, up from only 5,273 cases in 2016.

… the sheer dominance of disinformation in science and medicine should disabuse us of the comfortable notion this is something we can disregard…

A similar cocktail of scaremongering claims spread about the HPV vaccine in Japan saw uptake collapse from 70 to 1% in months, a deplorable situation estimated to cost upwards of 11,000 lives (Simms *et al*, 2020). Anti‐vaccine propaganda also markedly pushed down rates in Denmark and Ireland, a situation only being reversed in the latter country thanks to Herculean efforts by patient advocates, scientists, and physicians. Exposure to ubiquitous online anti‐vaccine conspiracy theories has, perhaps unsurprisingly, substantial impact on parental intention to vaccine (Jolley & Douglas, 2014): Faced with a plethora of worrying claims and unsure what to believe, apprehensive parents frequently avoid immunisation. Even now, as researchers try to derive a vaccine for the novel coronavirus, anti‐vaccine campaigners have already begun to spread falsehoods suggesting that trial participants are dying.

The dark renaissance of conquerable diseases is accordingly symptomatic of a deeper problem: The triumph of emotive fictions over reality. False narratives have an alluring simplicity for believers, but they do substantial harm; the staggering popularity of false or unsubstantiated cancer cures is but one example. Social media is rife with dubious claims: In 2016, more than half of the 20 most shared articles about cancer on Facebook involved medically discredited claims. Purveyors of false remedies accuse the medical and scientific community of suppressing cures for cancer, despite this being totally implausible (Grimes, 2016). This sadly cannot be dismissed a mere fringe belief: A staggering 37% of Americans believe the FDA is doing precisely this.

These claims of alternative treatments are at best useless, but they do harm far beyond their intrinsic lack of efficacy. When effective interventions such as radiotherapy and chemotherapy are dismissed as “poisons” and alternative or unsubstantiated treatments touted in their stead, it comes at terrible cost. Evidence suggests patients who subscribe to alternative approaches are more than twice as likely to die in the same period as those relying on conventional therapies (Johnson *et al*, 2018). The dark irony is that such conspiratorial narratives induce an inherent distrust of conventional medicine, providing a cloak for charlatans to operate under. Some common reasons for the perpetuation of health conspiracy are given in Box.

**Box 1. Some motivations for perpetuating health conspiracy**

*Epistemic:* Health conspiracies offer simple narratives for complex phenomena and provide a perverse sense of security against uncertainty for believers (Douglas *et al*, 2017).

*Egotistic:* More narcissistic individuals are more likely to be conspiracy theorists, and the illusion of special knowledge rendering one superior to others can be alluring (Douglas *et al*, 2017; Imhoff & Lamberty, 2017).

*Political:* Some health conspiracies have political dimension, when their propagation is deemed damaging to an enemy power by undermining public trust.

## Availability heuristics, illusory truth, and dubious amplification

It is crucial to note that this is problem that affects us all, and not a simple function of intelligence or education. Evidence suggests that we are collectively poor at differentiating between sources reputable and reprehensible. Compounding the noxious influence of conspiracy theory, it also persists longer and diffuses much further than reliable medical and scientific information. We can also fall victim to the phenomenon of illusory truth, where mere repetition of a claim induces us to accept it more readily, even if we know it to be false on an intellectual level. The availability heuristic plays an important role too—we afford more weight to easily recalled assertions than more sober‐headed analysis, and it is unsurprising that frightening stories perpetuate wider than more careful claims.

As humans, we emote first and reflect after; this propensity to react before reflecting is a trojan horse for devious fictions to become established. Social media, of course, cannot be blamed entirely for this phenomenon of “dubious amplification” (Grimes *et al*, 2020)—any media magnification of an unsubstantiated claim on any platform can cause harm. To take but one contemporary illustration, US President Donald Trump’s promotion of hydroxychloroquine for COVID‐19 led to a massive increase in use around the world, despite a paucity of evidence for efficacy, and emerging data pointing to severe harms. But while dubious amplification is a ubiquitous problem on all platforms, social media is unparalleled in its capacity to distort. A 2018 study in *Science* (Vousoughi et al, 2018) analysed 126,000 contested news stories between 2006 and 2017, with the alarming conclusion that hoax and rumour completely eclipse truth, and that fictions consistently dominate the narrative.

But while dubious amplification is a ubiquitous problem on all platforms, social media is unparalleled in its capacity to distort.

## Information hygiene and critical thinking

Long term, the only lasting vaccine against this onslaught of poisonous fictions is to improve our societal critical‐thinking skills. If there is a silver‐lining to COVID‐19, it might be that it has focused the world’s attention upon the vital importance of physical hygiene. The importance of rigorous hand‐washing and social distancing to stem the tide of the virus is something we collectively understand. But in tandem with this, we need to recognise that health disinformation goes viral too and does serious harm. If we are to protect ourselves against the ramifications of these rapidly increasing fictions, we must practice a new protective measure, a form of Informational Hygiene. Rather than risk unprotected exposure, we need to recognise that a healthy scepticism is our best protection against damaging fictions about our health.

Long term, the only lasting vaccine against this onslaught of poisonous fictions is to improve our societal critical‐thinking skills.

We must recognise that exposure to misinformation is harmful, take precautions to prevent ourselves becoming infected, and avoid vectors of misinformation in the sound and fury of bogus health claims. Before we perpetuate claims we encounter, we ought to ask ourselves whether they are reputable and verified before we infect others. In much the same way, we take precautions with potential pathogens, and it is crucial that we become aware that misinformation and conspiracy theory are not harmless: Even if it does not affect us badly, it may put others at serious risk.

## A wider responsibility

But the onus for this cannot solely be placed at the feet of the public—social media companies cannot shirk their responsibility for the deplorable situation in which we now find ourselves. In the last few months, companies such as Facebook, YouTube, and Instagram have pledged to crack down on everything from anti‐vaccine propaganda to COVID‐19 conspiracy theories and 5G scaremongering. But it is hard to interpret this as anything more than shallow lip‐service, as dangerous disinformation remains dominant across these channels. The most charitable interpretation is that social media companies are completely ineffectual at counteracting the volume of dangerous nonsense hosted on their platforms, or so inept as to not recognise it. A more cynical reading is that they simply do not care—to social media companies all engagement is profit, and the consequences for public health and understanding utterly inconsequential to them.

Whatever the reason, it is clear is that we cannot rely on social media giants to self‐regulate their platforms, despite their implorations to the contrary. How we best counteract the dangerous reality we now find ourselves in is not immediately obvious. It is a problem that demands more research, as we are sorely in need of evidence‐based policy to counteract the dangerous fictions to which we are constantly exposed, before they do further harm. Regulation of social media is a solution which must be seriously considered, and legislation that compels them to enforce fact‐checking and remove dubious content is perilously overdue. However, this is likely to take time, and given the sheer size of these giants, unrelenting international pressure. In the interim, we must acknowledge that it remains an environment thoroughly contaminated with viral misinformation, and basic information hygiene is the only means we must protect ourselves and each other. Pandora's box is already open—it is imperative now we learn to mitigate the consequences.

Regulation of social media is a solution which must be seriously considered…

**Further Reading**

Attitudes to COVID conspiracy. I would also then add this BBC article: https://www.bbc.com/news/technology-53083341

**The Infodemic**

World Health Organization (2020). Novel coronavirus (2019‐nCoV). Situation Report, 28

**Illusory truth and the spread of disinformation**

Brady WJ , Wills JA , Jost JT , Tucker JA , Van Bavel JJ (2017) Emotion shapes the diffusion of moralized content in social networks. Proc Natl Acad Sci USA 114: 7313–7318 2865235610.1073/pnas.1618923114PMC5514704

Del Vicario M , Bessi A , Zollo F , Petroni F , Scala A , Caldarelli G , Stanley HE , Quattrociocchi W (2016) The spreading of misinformation online. Proc Natl Acad Sci USA 113: 554–559 2672986310.1073/pnas.1517441113PMC4725489

Fazio LK , Brashier NM , Payne BK , Marsh EJ (2015) Knowledge does not protect against illusory truth. J Exp Psychol Gen 144: 993 2630179510.1037/xge0000098

Grimes DR (2020)The irrational ape ‐ why flawed logic puts us all at risk and how critical thinking can save the world, 1st edn London: Simon & Schuster

Johnson NF , Velásquez N , Restrepo NJ , Leahy R , Gabriel N , El Oud S , Zheng M , Manrique P , Wuchty S , Lupu Y (2020) The online competition between pro‐and anti‐vaccination views. Nature 1–4 10.1038/s41586-020-2281-132499650
